# Efficacy and Safety of Hou Gu Mi Xi on Spleen Qi Deficiency in Patients with Nonorganic Gastrointestinal Disorders: Protocol for a Multicenter, Randomized, Placebo-Controlled Trial

**DOI:** 10.1155/2018/1980491

**Published:** 2018-12-02

**Authors:** Xiaofan Chen, Heyun Nie, Wenjun Liu, Xu Zhou, Jianhua Nie, Bin Xie, Dongping Chen, Yiping Jiang, Kunhe Zhang, Ying Fu, Deping Yang, Yan Xiong, Zhangyang Zhao, Xin Sun, Weifeng Zhu

**Affiliations:** ^1^Evidence-Based Medicine Research Center, School of Basic Medical Sciences, Jiangxi University of Traditional Chinese Medicine, Jiangxi, China; ^2^School of Food Science and Engineering, Inner Mongolia Agricultural University, Inner Mongolia, China; ^3^Department of Spleen, Stomach, Liver and Gallbladder Diseases, The Affiliated Hospital of Jiangxi University of Traditional Chinese Medicine, Jiangxi, China; ^4^Department of Gastroenterology, The First Affiliated Hospital of Nanchang University, Jiangxi, China; ^5^Department of Traditional Chinese Medicine, The Second Affiliated Hospital of Nanchang University, Jiangxi, China; ^6^Department of Gastroenterology, Nanchang Hospital of Integrated Traditional Chinese and Western Medicine, Jiangxi, China; ^7^Department of Internal medicine, Nanchang Hongdu Hospital of Traditional Chinese Medicine, Jiangxi, China; ^8^Chinese Evidence-Based Medicine Center, West China Hospital, Sichuan University, Sichuan, China

## Abstract

**Background:**

There is a worldwide epidemic of nonorganic gastrointestinal disorders (NOGDs), which are a class of disorders that cause various discomforts and ultimately progress into organic gastrointestinal diseases. Because of the unsatisfactory efficacy of Western medical treatments, traditional Chinese medicine (TCM) is becoming a promising complementary and alternative treatment to manage NOGDs.

**Objectives:**

To investigate the efficacy and safety of Hou Gu Mi Xi (HGMX), a newly developed dietary TCM formula, on the syndrome of spleen qi deficiency (SQD) in patients with NOGDs.

**Methods/Design:**

This study is a multicenter, randomized, double-blinded, parallel, and placebo-controlled trial that will last for 2 years. All qualified subjects with NOGDs and SQD will be included. The study population will be divided into the HGMX and placebo groups. To assess the efficacy of HGMX, we will mainly focus on changes in SQD symptoms scored by a Spleen Qi Deficiency Symptoms Grading and Quantifying Scale and evaluate changes in gastrin-17, the negative* Helicobacter pylori* conversion rate, body weight, body mass index, and gastroscopy findings. The safety of HGMX will be assessed by recording adverse events (AEs), severe AEs, treatment-related AEs and withdrawal due to AEs.

**Discussion:**

This trial is part of our study series that intends to validate the potential of HGMX in the management of chronic gastrointestinal diseases. This series of RCTs is the first committed to the evaluation of a dietary TCM formula and will hopefully establish an evidence-based clinical research model for dietary TCM formulas.

**Ethics:**

The protocol was approved by Ethics Committee of five research hospitals and was registered in Clinicaltrials.gov (NCT03019042).

## 1. Introduction

Chronic gastrointestinal diseases are one of the major health problems worldwide, with an incidence of approximately 60 to 70 million cases per year in the United States [1]. It was reported that up to 38.6 million clinic visits and 45.3 deaths per 100 thousand patients per year are due to chronic gastrointestinal diseases [1, 2]. Meanwhile, the incidence of chronic gastrointestinal diseases in China is 7.3‰, ranking fifth among all diseases [3]. Pathologically, chronic gastrointestinal diseases can be divided into organic gastrointestinal diseases and nonorganic gastrointestinal disorders (NOGDs); organic gastrointestinal diseases can develop from NOGDs, and a typical routine of pathological progression is from NOGDs to gastrointestinal metaplasia, atypical hyperplasia, precancerous lesions, and, ultimately, cancer [4, 5]. Therefore, although explicit epidemiological data for NOGDs remain unclear, the harm and burden of NOGDs are plausibly substantial.

NOGDs represent a series of disorders, mainly including chronic nonatrophic gastritis and functional gastrointestinal disorders listed in the Rome IV diagnostic criteria [6]. The symptoms of NOGDs are nontypical and may include gastrointestinal discomfort, such as nausea, stomach and abdominal distension, vomiting, acid reflux, diarrhea, and constipation, as well as systemic symptoms [6, 7]. Because it is challenging to cure most organic gastrointestinal diseases, such as atrophic gastritis, peptic ulcers, and gastric cancer, people are focusing on treating NOGDs and preventing progression, which would be more cost-effective than treating organic gastrointestinal diseases [8, 9]. However, the efficacy of most treatment options, predominantly anti-infection, anti-inflammation, and symptomatic treatments, in current Western medicine guidelines for NOGDs is unsatisfactory [10, 11].

Along with the development of medical science, traditional Chinese medicine (TCM) is playing an increasingly important role in the treatment of chronic gastrointestinal diseases, especially NOGDs [4]. The common symptoms of NOGDs mentioned above can be categorized as six syndromes in TCM—spleen qi deficiency (SQD), spleen-stomach deficiency cold, spleen deficiency and dampness, spleen deficiency and liver depression, liver and spleen imbalance, and liver qi stagnation—among which SQD is the most common, and the primary symptoms include stomach and abdominal distension, fatigue, weakness, loss of appetite, and diarrhea [12]. Spleen and qi are concepts in TCM that play roles in transiting food and water and maintaining activity, respectively. The TCM-based pathogenesis of spleen qi-deficient NOGDs is dysfunction in the spleen and stagnation of qi, which cause blood stasis, accumulation of phlegm, and food retention. Therefore, TCM therapy for NOGDs aims to invigorate and replenish qi, supported by nourishing the spleen, activating blood circulation, and removing damp evil, qi stagnation, and food retention [13].

Shen Ling Bai Zhu San, a classic Chinese herbal medicine formula, was originally described in* Tai Ping Hui Min He Ji Ju Fang* in the Nang Song Dynasty (1102 AD) [14]. Because of its effects of replenishing qi and nourishing the spleen, it has been used to treat SQD for thousands of years, especially for patients with NOGDs [15]. However, Shen Ling Bai Zhu San is a poor tasting powder that must be mixed with boiled water, which restricts its widespread use and long-term compliance.

On the basis of Shen Ling Bai Zhu San, a dietary TCM formula named Hou Gu Mi Xi (HGMX) is under development by Jiangzhong Pharmaceutical (group) Co., Ltd., Jiangxi, China, and had come into the market in September 2016, in which atractylodes (an herb that is forbidden in food) is replaced with orange peel. Consequently, HGMX is composed of ginseng (*Renshen*), tuckahoe (*Fuling*), coixenolide (*Yiyiren*), Chinese yam (*Shanyao*), lotus seed (*Lianzi*), amomum (*Sharen*), platycodon (*Jiegen*), white hyacinth bean (*Baibiandou*), licorice (*Gancao*), and orange peel (*Jupi*), all in the list of food in China. In this formula, ginseng, tuckahoe, coixenolide, and Chinese yam can invigorate spleen qi, remove dampness, and nourish our body, with the support of lotus seed, amomum, platycodon, white hyacinth bean, licorice, and orange peel. Theoretically, HGMX can replenish the spleen and stomach qi, remove the dampness and turbidity, and dissipate the qi stagnation such that the spleen qi can run normally and the damp evil can be removed, leading to the natural elimination of the symptoms of SQD. In addition, HGMX has been made into an oatmeal preparation and can be easily transformed into a rice paste with boiled water and can become our everyday breakfast. As a dietary formula, HGMX should be safe and show improved compliance with long-term intake.

However, the efficacy, safety, and compliance of HGMX are based solely on scientific theories and hypotheses, which are still under inspection. Therefore, we plan to conduct a postmarket, hospital-based, randomized controlled trial (RCT) to investigate the long-term efficacy and safety of HGMX on SQD in patients with NOGDs.

## 2. Methods/Design

### 2.1. Trial Design

**Figure 1:**
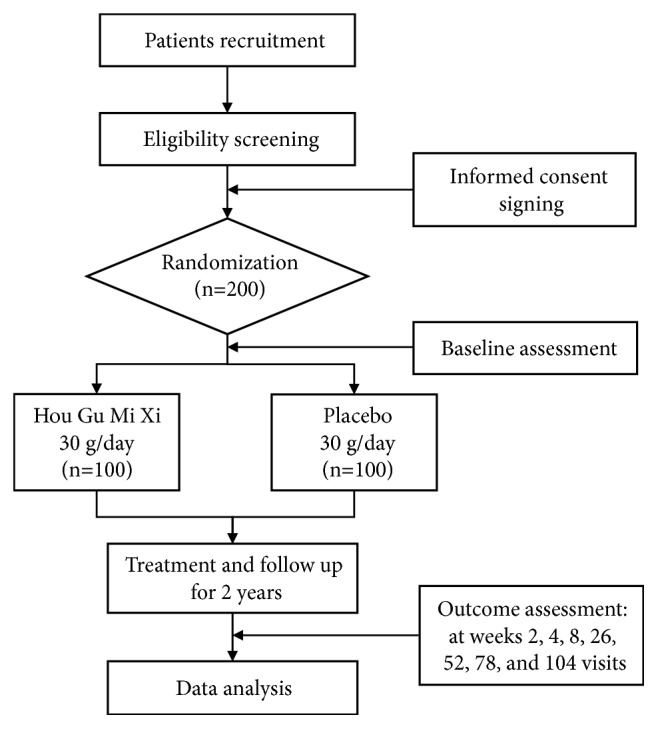
Overall design of the study.

This study is a multicenter, randomized, double-blinded, parallel-group, placebo-controlled trial that aims to determine whether HGMX is an effective and safe treatment for improving the symptoms of SQD in patients with NOGDs. The study population will be randomly divided into the HGMX group and the placebo group. The overall design is presented in Figure 1. The trial has been registered at Clinicaltrials.gov (No. NCT03019042). In order to guarantee standardized processes, precise judgment, and the safety of all participants, our study will be performed in accordance with the principles of Good Clinical Practices and the Declaration of Helsinki. We designed and reported our protocol according to Standard Protocol Items: Recommendations for Interventional Trials. [16].

### 2.2. Sites and Participant Recruitment

The potential participants will be recruited in three ways: oral promotion by researchers, hospital posters, and web page publicity. Patients with NOGDs will be recruited from five tertiary hospitals: First Affiliated Hospital of Nanchang University, Second Affiliated Hospital of Nanchang University, Jiangxi Hospital of Traditional Chinese Medicine, Nanchang Hospital of Integrated Traditional Chinese and Western Medicine, and Nanchang Hongdu Hospital of Traditional Chinese Medicine. Subjects who meet the inclusion criteria will be required to sign the informed consent form to show an overall understanding of study details prior to study participation.

### 2.3. Inclusion Criteria

Participants meeting all the following criteria will be included:

1. Diagnosis with NOGDs by gastroscopy based on the Consensus on Integrated Traditional Chinese and Western Medicine in the Treatment of Chronic Gastritis and the ROMA IV criteria [6, 7], primarily including chronic nonatrophic gastritis, functional dyspepsia, and irritable bowel syndrome
2. Diagnosis with SQD status according to the criteria in the Clinical Guidelines of New Drugs for Traditional Chinese Medicine [13]
3. At least 14 years old
4. Sign the informed consent form

### 2.4. Exclusion Criteria

Participants who report any of the following conditions will be excluded:

1. Patients who have gastrointestinal disorders with organic pathologic changes, such as peptic ulcers, gastrointestinal erosions, gastroesophageal reflux disease, gastrointestinal hemorrhage or perforation, structural changes in gastrointestinal structure, gastrointestinal vascular diseases, ileus, or a benign tumor
2. Pregnant or breast-feeding women
3. Patients who have allergic history to the sample or sample components
4. Patients with impaired liver or kidney function, including one of the following conditions: (a) total bilirubin, alanine transaminase, or aspartate aminotransferase > 2 upper limit of normal (ULN); (b) serum creatinine >2 ULN
5. Patients with an obviously abnormal electrocardiogram (ECG), severe mental disorders, or any diagnosed malignancies;
6. Patients who have taken drugs that can damage the stomach and intestine, such as nonsteroidal anti-inflammatory drugs, theophylline, oral antibiotics, or potassium supplements within 3 months
7. Patients who have received any agents or other intervention for his/her gastrointestinal disorder within 3 months
8. Patients who are unwilling to provide personal information or who cannot understand and sign the informed consent form

### 2.5. Randomization and Blinding

We used computer-based statistical analysis software to generate a randomization system that is saved as enciphered data. For allocation concealment, participants are grouped with a centralized randomization assignment. Except in cases of a severe adverse event (SAE), all the participants, medical care providers, data collectors, and outcome reviewers will be blinded before the data analysis. Hence, we established a randomization management center that is in charge of all processes related to random sequence generation, allocation concealment, and blinding. As a result, all the members of this center were excluded from any other processes in this study.

### 2.6. Interventions

Our participants are grouped into the HGMX group (experimental group) and the control group. In the HGMX group, participants will be treated with an oral dose of 30 g/day HGMX. Every 30 g of HGMX contains 10.1 g of herbal material (detailed components of HGMX are shown in Table 1) and 19.9g early rice and oats. Participants in the control group will receive the same dose of placebo, which consists of 66.7% early rice and 33.3% oats with the same preparation, packaging, labeling, and appearance but a slightly different taste and smell than the real HGMX. All subjects will be treated for a duration of 2 years (104 weeks). During follow-up, patients can be treated as necessary based on clinical guidelines for acute and chronic diseases (such as hypertension, diabetes, and osteoarthritis) that are not included in the exclusion criteria.

### 2.7. Outcomes

#### 2.7.1. Primary Outcome

The primary outcome is the change in the score of the Spleen Qi Deficiency Symptoms Grading and Quantifying Scale (SQD scale), which was derived from a classic scale in the Clinical Guidelines of New Drugs for Traditional Chinese Medicine to Validate Spleen Qi Deficiency Symptoms [13]. The SQD scale consists of 17 items that assess (1) stomach distension, (2) abdominal distension, (3) physical fatigue and weakness, (4) mental fatigue and taciturnity, (5) loss of appetite, (6) abnormal stools, (7) stomach pain, (8) stomach tightness, (9) abdominal pain, (10) acid reflux, (11) belching, (12) nausea and vomiting, (13) abnormal bowel sounds, (14) powerless defecation, (15) sallow complexion, (16) loss of taste and hypodipsia, and (17) facial and limb edema. Items 1-6 focus on primary symptoms, and the other items focus on secondary symptoms. The scale assesses all the symptoms of SQD, including the duration of each episode (0-3 points, available for items 1-2 and 7-9), the severity (0-3 points, not available for item 13), and the number of episodes per day (0-4 points, except 0-3 for item 5) and per week (0-5 points). The score of each item will be used to calculate the total score, with 2 points for primary symptoms and 1 point for secondary symptoms. As a result, the total score ranges from 0 to 292, and higher scores indicate more severe symptoms. Compared with the original scale, we have added four symptoms (items 1, 7, 10, and 11) and defined 5 primary symptoms. In addition, we assess not only the severity, which is included in the former scale, but also the duration and the number of episodes per day and per week for these symptoms. For more details, see Table S1 in the supplementary material.

#### 2.7.2. Secondary Outcomes

- Changes in gastrin-17 (ng/L) levels in order to determine whether the intervention can improve gastric function were observed
- Changes in body weight (kg) and body mass index (kg/m^2^) were observed
- Quantitative changes were observed by gastroscopy

#### 2.7.3. Safety Outcomes

- Incidence of adverse events (AEs), including abnormal ECG and dramatic changes (indicated by a value outside 2 × the lower or upper normal reference interval) in alanine transaminase (U/L), aspartate aminotransferase (U/L), total bilirubin (*μ*mol/L), direct bilirubin (*μ*mol/L), indirect bilirubin (*μ*mol/L), or serum creatinine (*μ*mol/L). In addition, doctor-observed and patient-reported AEs are also included.
- SAEs are those AEs that could result in treatment in the hospital, a longer in-hospital stay, transfer to an intensive care unit, disability, life-threatening complications, and death.
- Treatment-related AEs defined as those AEs fully meet the following condition [17]: (1) the occurrence time is logically related to treatment administration; (2) the adverse effects cannot be interpreted by cointerventions and comorbidities; (3) the adverse effects should relieve or disappear when the treatment is withdrawn; (4) the relation is supported by definitively pharmacological or phenomenological mechanism.
- Withdrawal was due to AEs.

### 2.8. Study Follow-Up and Outcome Assessments

The durations of follow-up and treatment are equal at 2 years. In our study, all participants will be evaluated 8 times: at baseline and in weeks 2, 4, 8, 26, 52, 78, and 104. After the qualified participants sign the informed consent form, they will document the characteristics of their own symptoms in the baseline case report form (CRF) and then record the baseline (first visit) physical and laboratory examination data. In weeks 2 and 4, participants will complete the Spleen Qi Deficiency Symptoms Grading and Quantifying Scale (SQD scale) and a physical examination. In weeks 8, 26, 52, 78, and 104, an ECG and laboratory examination, including routine blood, urine, and stool tests, are required. Gastrin-17 and* Helicobacter pylori* will be assessed at both weeks 52 and 104. Gastroscopy will be performed at only baseline and the last visit.  Table 2 provides an overview of the study procedures.

### 2.9. Sample Size Estimation

This RCT is a superiority study. From an overall consideration, we will restrict the type I error (*α*) and type II error (*β*) to 0.05 and 0.2, respectively. The variation between groups and the combined standard deviation of the primary outcome were estimated at 6.0 and 13.0, respectively, based on the results of a preliminary observational study. In addition, a maximum rate of 20% withdrawal or loss to follow-up was adopted. Therefore, with the corresponding sample size estimation equation [18] and the consideration of funding constraints, we computed an expected sample size of 200, with 100 in each group.

### 2.10. Statistical Analysis

We will conduct an interim analysis at week 52 and a final analysis at week 104 with the help of the statistical software SPSS v23.0.

Specifically, an intention-to-treat analysis will be adopted for primary outcomes, while the per-protocol analysis will be performed as a sensitivity analysis. The last observation carried forward method will be used for missing data due to loss to follow-up. Continuous and categorical variables will be analyzed by t-test (or analysis of variance) and Pearson chi-squared test, respectively. In addition, continuous variables with a skewed distribution will be compared with the nonparametric rank-sum test. A P value ≤ 0.05 will indicate statistical significance for all the comparisons mentioned above, and group sequential method will be used to adjust the significant boundary for multiple t-test [19]. Subgroup analysis stratified by type of NOGDs (nonatrophic gastritis versus functional gastrointestinal disorders) and infection of* Helicobacter pylori* (yes versus no) will be performed. Furthermore, an analysis based on safety data set with no imputation will also be conducted. The incidence of AEs in the two groups will be compared using the Pearson chi-squared test, and all the AEs will be described in detail.

### 2.11. Data Collection and Management

Our data collection and management will be carried out under collaboration with our research associates, coordinators, and doctors. All the investigators will undergo standard operating procedures training, in which the follow-up, data collection and management should be strictly performed. The procedures and results of the SQD scale scores, vital sign measurements, sample collection, and laboratory examinations will be performed and obtained under the control and supervision of doctors.

Our research coordinators are college student volunteers from Jiangxi University of Traditional Chinese Medicine who have the responsibilities of contacting patients, helping patients and doctors communicate, and checking data and cross-entry. Meanwhile, several research associates in charge of standard performance, patient compliance, completeness of data collection, and problem solving will be selected from researchers at Jiangxi University of Traditional Chinese Medicine. We will ensure that each study center has a research associate.

The confidentiality of primary CRFs will be protected after data entry. The computerized database will be saved with double password protection and exclusively used for data analysis, in which patient information will be processed anonymously. Only statistical analysis staff will have access to our database, and they will be excluded from procedures other than data analysis.

### 2.12. Patient Compliance

To enhance patient compliance, the following measures will be adopted: (1) we will fully assess patients' compliance at the stage of eligibility screening to exclude patients with the consideration of poor compliance; (2) we will provide a traffic subsidy (100 China Yuan) to the participants for each follow-up. (3) A 3-day deviation in follow-up will be accepted at weeks 2, 4, and 8, and a 7-day deviation will be acceptable at weeks 26, 52, 78, and 104. (4) We will establish a WeChat (a smartphone application) group to keep in online touch with the patients and provide health counselling services to improve patients' enthusiasm. (5) For all samples allocated to patients, a recycling check will be conducted to ensure patient compliance and to confirm the correct intake dose.

### 2.13. Discontinuation and Withdrawal

After the assessment of previous compliance, study of the corresponding participant will be discontinued for the following reasons, and the participant will be withdrawn without laboratory examination.

- The symptoms of SQD worsen and the patient must be treated with an alternative regimen to improve his/her status
- The SAEs occurred. All SAEs will be reported by our principle investigators to the ethics committee of the local hospital and the Jiangxi Food and Drug Administration Agency within 24 hours. Several emergency measures will be taken to minimize the harm to patients when a SAE occurred. We will immediately assign senior doctors to implement rescue measures according to the corresponding guidelines. If acute toxicity caused by overdose is determined, the doctors will give measures to accelerate the drug excretion, and, if necessary, the intensive care (e.g., electrocardiograph monitoring and assisted respiration) will be performed until patients' vital signs are stable. If the patient is unable to come to the research hospital, we will advise him/her to visit the local hospital and give consultation with the local doctors, and, if necessary, we will appoint senior doctors to the local hospital
- The patient decides to take prohibited drugs to improve NOGDs
- Poor compliance includes an intake dose less than 80% of that required or two follow-ups beyond the specific deviation range
- Participant has personal concerns

## 3. Discussion

Since the 20th century, the disease spectrum has undergone significant changes: the chronic diseases, such as nonorganic disorders, diabetes mellitus, cardiovascular diseases, and cancer, become the major disease burden. The development of these diseases is multifactorial and the diet often plays an important role. Thus, diet-related management (e.g., diet control and nutrition supplement) could be a treatment approach for these diseases. However, the clinical nutriology simply based on adjustment of diet structure has limitations on the efficacy.

Using the TCM theory, the food (including herbs in the list of food) can be made into dietary TCM formula, which can be daily eaten and has treatment effects for chronic diseases. HGMX is a dietary TCM formula developed based on this concept. After screening of a large number of classic TCM formula, Shen Ling Bai Zhu San was chosen as the basic formula of HGMX, because its efficacy and safety for gastrointestinal diseases are validated by thousands of years of application and animal and clinical researches. On the other hand, except for atractylodes, all components of Shen Ling Bai Zhu San are in the official list of food designated by the Ministry of Health of China. Thus, it can be easily modified to a pure dietary formula on the premise without changing the main effects. During the two years of marketing, no adverse events related to HGMX are reported. Therefore, the safety of this RCT can also be guaranteed.

To validate the potential effects of HGMX on gastrointestinal protection and systemic symptom improvement, we intend to perform a series of RCTs. The study series contains 3 RCTs, with the other two focusing on patients with peptic ulcers (NCT03320538) and on patients who underwent radical gastrectomy for gastric cancer (NCT03025152). These RCTs are also corresponding to three main applications of dietary TCM formula: (1) use for the treatment of nonorganic disorders; (2) adjuvant therapy for the treatment of chronic diseases; and (3) adjuvant therapy for the rehabilitation of major diseases.

The main purpose of this RCT is to clarify the long-term efficacy, safety, and compliance of HGMX, as a dietary TCM formula, in patients with SQD and NOGDs, with the guidance of TCM theory and evidence-based medicine methodology. The randomization of this RCT is standardly designed to minimize the selection bias and the period of follow-up is enough to observe the long-term efficacy and safety. Before the beginning of the trial, we appointed a training group contained senior methodologists, pharmacists, and physicians to conduct a standard operation procedure training in each research hospital. The diagnosis of TCM syndrome and the measurement of patient-reported outcomes will be performed by well-trained TCM physicians according to the standards to increase the consistency between difference investigators and hospitals. We also prespecified two sets of subgroup analysis based on expert's opinion and biological mechanism and used the group sequential method in repeated measures to reduce the probability of type I error. The results of the trial will be reported in strict accordance with the checklist of Consolidated Standards of Reporting Trials (CONSORT) Extension for Chinese Herbal Medicine Formulas. Therefore, this RCT can be expected to produce high-quality evidence.

This study may have some limitations. First, we will use an adjusted scale to assess SQD symptoms without reliability and validity tests, which could introduce a detection bias. We will conduct reliability and validity tests based on our study outcome data to test whether there exist any defects in reliability and validity. Second, because the placebo consists of early rice and oats and will be undertaken regularly according the study protocol, the patients in the placebo group may correct some bad diet habits during the participation, which may have positive effects on NOGDs and lead to bias in results (i.e., the estimate may tend to be conservative). Third, although we will take various measures to improve the patient compliance, the loss to follow-up is still probably inevitable during the long-term follow-up, resulting in attrition bias.

The dietary TCM formula is currently becoming a hot concern in the area of TCM owing to the safety attribute of diet and the therapy attribute of herb. However, the incomplete industry standards and evaluation methodology are restraining the development and application of the dietary TCM formula. A critical reason of these problems is the extreme scarcity of high-quality evidence. Therefore, using rigorous methodology to evaluate efficacy and safety of the dietary TCM formula in the framework of evidence-based medicine is a necessary approach to address these issues. We hope to take the first step to produce high-quality evidence for the dietary TCM formula by carrying out this RCT series, which will establish a reference for the future clinical researches of other dietary TCM formulas and will be of important value in developing related regulations.

## Figures and Tables

**Table 1:** Detailed components of Hou Gu Mi Xi.

Component	Part used	Proportion
Renshen (ginseng)	Root	1.67%
Fuling (tuckahoe)	Sclerotium	6.67%
Yiyiren (coixenolide)	Seed	6.67%
Shanyao (Chinese yam)	Root	3.33%
Lianzi (lotus seed)	Fruit	3.33%
Sharen (amomum)	Fruit	0.33%
Jiegen (Platycodon)	Stem	1.67%
Baibiandou (white hyacinth bean)	Seed	6.67%
Gancao (licorice)	Stem	1.67%
Jupi (orange peel)	Pericarp	1.67%
Early rice	Seed	43.22%
Oats	Seed	23.10%

**Table 2:** Procedures of the trial.

Item	Baseline	2^nd^ week	4^th^ week	8^th^ week	26^th^ week	52^th^ week	78^th^ week	104^th^ week
Eligibility screening	√							
Signed inform consent	√							
Spleen Qi Deficiency Symptoms Grading and Quantifying Scale	√	√	√	√	√	√	√	√
Physical examination (height, weight, blood pressure, heart rate)	√	√	√	√	√	√	√	√
Blood, urine, and stool routine examination	√			√	√	√	√	√
Electrocardiogram	√			√	√	√	√	√
Liver function (ALT, AST, TBIL, DBIL, IBIL)	√			√	√	√	√	√
Kidney function (Scr, BUN)	√			√	√	√	√	√
Gastric function (Gastrin-17)	√					√		√
Helicobacter Pylori	√							
Gastroscopy	√							√
Record of adverse events				√	√	√	√	√
Compliance assessment		√	√	√	√	√	√	√

ALT = alanine transaminase, AST = aspartate aminotransferase, TBIL = total bilirubin, DBIL = direct bilirubin, IBIL = indirect bilirubin, SCr = serum creatinine, BUN = urea nitrogen, and PT= prothrombin time.

## Data Availability

No data were used to support this study.

## Abbreviations

AEs: Adverse events
ALT: Alanine aminotransferase
APTT: Activated partial thromboplastin time
AST: Aspartate aminotransferase
BUN: Blood urea nitrogen
BUN: Urea nitrogen
DBIL: Direct bilirubin
HGMX: Hou Gu Mi Xi
IBIL: Indirect bilirubin
NOGDs: Nonorganic gastrointestinal disorders
RCT: Randomized controlled trial
SAEs: Severe adverse events
SCr: Serum creatinine
SQD: Spleen qi deficiency
SQD scale: Spleen Qi Deficiency Symptoms Grading and Quantifying Scale
TBIL: Total bilirubin
CM: Traditional Chinese medicine
ULN: Upper limit of normal.

## Supplementary Materials

Supplementary Materials Table S1. Spleen Qi Deficiency Symptoms Grading and Quantifying Scale.
