# Supplementary material for: Efficacy and Safety of Hou Gu Mi Xi on Spleen Qi Deficiency in Patients with Nonorganic Gastrointestinal Disorders: Protocol for a Multicenter, Randomized, Placebo-Controlled Trial
Source: Evid Based Complement Alternat Med. 2018 Dec 2;2018:1980491. doi: 10.1155/2018/1980491 (PMC6304527; doi:10.1155/2018/1980491)
Supplement: Supplementary Materials — Table S1. Spleen Qi Deficiency Symptoms Grading and Quantifying Scale. [file 1980491.f1.docx]

**Table S1. Spleen Qi Deficiency Symptoms Grading and Quantifying Scale**

| **Item** | **Option** |
| --- | --- |
| **1 Stomach distension^*^** | □_0_ None |
| Duration | □_1_ < 0.5 hour □_2_ 0.5-1 hour □_3_ > 1 hour |
| Severity | □_1_ Mild □_2_ Moderate □_3_ Severe |
| Frequency per day | □_1_ Occasionally　□_2_ Sometimes　□_3_ Most of the time　□_4_ Persistently |
| Frequency per week | □_1_ < 1 day □_2_ 1 day □_3_ 2-3 days □_4_ 4-5 days □_5_ 6-7 days |
| **2 Abdominal distension^*^** | □_0_ None |
| Duration | □_1_ < 0.5 hour □_2_ 0.5-1 hour □_3_ > 1 hour |
| Severity | □_1_ Mild □_2_ Moderate □_3_ Severe |
| Frequency per day | □_1_ Occasionally　□_2_ Sometimes　□_3_ Most of the time　□_4_ Persistently |
| Frequency per week | □_1_ < 1 day □_2_ 1 day □_3_ 2-3 days □_4_ 4-5 days □_5_ 6-7 days |
| **3 Physical Fatigue and weakness^*^** | □_0_ None |
| Severity | □_1_ Mild □_2_ Moderate □_3_ Severe |
| Frequency per day | □_1_ Occasionally　□_2_ Sometimes　□_3_ Most of the time　□_4_ Persistently |
| Frequency per week | □_1_ < 1 day □_2_ 1 day □_3_ 2-3 days □_4_ 4-5 days □_5_ 6-7 days |
| **4 Mental fatigue and taciturnity**^*^ | □_0_ None |
| Severity | □_1_ Mild □_2_ Moderate □_3_ Severe |
| Frequency per day | □_1_ Occasionally　□_2_ Sometimes　□_3_ Most of the time　□_4_ Persistently |
| Frequency per week | □_1_ < 1 day □_2_ 1 day □_3_ 2-3 days □_4_ 4-5 days □_5_ 6-7 days |
| **5 Loss of appetite^*^** | □_0_ None |
| Severity | □_1_ Mild □_2_ Moderate □_3_ Severe |
| Frequency per day | □_1_ 1 meal □_2_ 2 meals □_3_ 3 meals |
| Frequency per week | □_1_ < 1 day □_2_ 1 day □_3_ 2-3 days □_4_ 4-5 days □_5_ 6-7 days |
| **6 Abnormal stools^*^** | □_0_ None |
| Severity | □_1_ Unshapen stool □_2_ Loose stool □_3_ Diarrhea |
| Frequency per day | □_1_ 1 time □_2_ 2 times □_3_ 3 times □_4_ ≥ 4 times |
| Frequency per week | □_1_ < 1 day □_2_ 1 day □_3_ 2-3 days □_4_ 4-5 days □_5_ 6-7 days |
| **7 Stomach pain** | □_0_ None |
| Duration | □_1_ < 0.5 hour □_2_ 0.5-1 hour □_3_ > 1 hour |
| Severity | □_1_ Mild □_2_ Moderate □_3_ Severe |
| Frequency per day | □_1_ Occasionally　□_2_ Sometimes　□_3_ Most of the time　□_4_ Persistently |
| Frequency per week | □_1_ < 1 day □_2_ 1 day □_3_ 2-3 days □_4_ 4-5 days □_5_ 6-7 days |

(*Continuous*)

| **Item** | **Option** |
| --- | --- |
| **8 Stomach tightness** | □_0_ None |
| Duration | □_1_ < 0.5 hour □_2_ 0.5-1 hour □_3_ > 1 hour |
| Severity | □_1_ Mild □_2_ Moderate □_3_ Severe |
| Frequency per day | □_1_ Occasionally　□_2_ Sometimes　□_3_ Most of the time　□_4_ Persistently |
| Frequency per week | □_1_ < 1 day □_2_ 1 day □_3_ 2-3 days □_4_ 4-5 days □_5_ 6-7 days |
| **9 Abdominal pain** | □_0_ None |
| Duration | □_1_ < 0.5 hour □_2_ 0.5-1 hour □_3_ > 1 hour |
| Severity | □_1_ Mild □_2_ Moderate □_3_ Severe |
| Frequency per day | □_1_ Occasionally　□_2_ Sometimes　□_3_ Most of the time　□_4_ Persistently |
| Frequency per week | □_1_ < 1 day □_2_ 1 day □_3_ 2-3 days □_4_ 4-5 days □_5_ 6-7 days |
| **10 Acid reflux** | □_0_ None |
| Severity | □_1_ Mild □_2_ Moderate □_3_ Severe |
| Frequency per day | □_1_ Occasionally　□_2_ Sometimes　□_3_ Most of the time　□_4_ Persistently |
| Frequency per week | □_1_ < 1 day □_2_ 1 day □_3_ 2-3 days □_4_ 4-5 days □_5_ 6-7 days |
| **11 Belching** | □_0_ None |
| Severity | □_1_ Mild □_2_ Moderate □_3_ Severe |
| Frequency per day | □_1_ Occasionally　□_2_ Sometimes　□_3_ Most of the time　□_4_ Persistently |
| Frequency per week | □_1_ < 1 day □_2_ 1 day □_3_ 2-3 days □_4_ 4-5 days □_5_ 6-7 days |
| **12 Nausea and vomiting** | □_0_ None |
| Severity | □_1_ Mild □_2_ Moderate □_3_ Severe |
| Frequency per day | □_1_ Occasionally　□_2_ Sometimes　□_3_ Most of the time　□_4_ Persistently |
| Frequency per week | □_1_ < 1 day □_2_ 1 day □_3_ 2-3 days □_4_ 4-5 days □_5_ 6-7 days |
| **13 Abnormal bowel sounds** | □_0_ None |
| Frequency per day | □_1_ Occasionally　□_2_ Sometimes　□_3_ Most of the time　□_4_ Persistently |
| Frequency per week | □_1_ < 1 day □_2_ 1 day □_3_ 2-3 days □_4_ 4-5 days □_5_ 6-7 days |
| **14 Powerless defecation** | □_0_ None |
| Severity | □_1_ Mild □_2_ Moderate □_3_ Severe |
| Frequency per day | □_1_ Occasionally　□_2_ Sometimes　□_3_ Most of the time　□_4_ Persistently |
| Frequency per week | □_1_ < 1 day □_2_ 1 day □_3_ 2-3 days □_4_ 4-5 days □_5_ 6-7 days |
| **15 Sallow complexion** | □_0_ None |
| Severity | □_1_ Mild □_2_ Moderate □_3_ Severe |
| Frequency per day | □_1_ Occasionally　□_2_ Sometimes　□_3_ Most of the time　□_4_ Persistently |
| Frequency per week | □_1_ < 1 day □_2_ 1 day □_3_ 2-3 days □_4_ 4-5 days □_5_ 6-7 days |

(*Continuous*)

| **Item** | **Option** |
| --- | --- |
| **16 Loss of taste and hypodipsia** | □_0_ None |
| Severity | □_1_ Mild □_2_ Moderate □_3_ Severe |
| Frequency per day | □_1_ Occasionally　□_2_ Sometimes　□_3_ Most of the time　□_4_ Persistently |
| Frequency per week | □_1_ < 1 day □_2_ 1 day □_3_ 2-3 days □_4_ 4-5 days □_5_ 6-7 days |
| **17 Facial and limb oedema** | □_0_ None |
| Severity | □_1_ Mild □_2_ Moderate □_3_ Severe |
| Frequency per day | □_1_ Occasionally　□_2_ Sometimes　□_3_ Most of the time　□_4_ Persistently |
| Frequency per week | □_1_ < 1 day □_2_ 1 day □_3_ 2-3 days □_4_ 4-5 days □_5_ 6-7 days |

*: primary symptoms

Reference for assessing severity of symptoms or signs:

Mild: The symptoms or signs are not obvious and patients cannot felt without reminding;

Moderate: The symptoms or signs are obvious but do not affect patients’ living and work;

Severity: The symptoms or signs are very obvious and affect patients’ living and work.
